# Mitochondrial genome of *Cossura pygodactylata* Jones 1956 (Annelida: Sedentaria) from the White Sea

**DOI:** 10.1080/23802359.2026.2670063

**Published:** 2026-05-11

**Authors:** Margarita Ezhova, Aleksandra Bezmenova, Vera Emelianenko, Anna Zhadan, Dmitry Knorre, Tatiana Neretina

**Affiliations:** a Faculty of Biology, N.A. Pertsov White Sea Biological Station, Lomonosov Moscow State University, Moscow, Russia; b Evolution, Cell Biology, and Symbiosis Unit, Okinawa Institute of Science and Technology, Onna, Japan; c Belozersky Institute of Physico-Chemical Biology, Lomonosov Moscow State University, Moscow, Russia

**Keywords:** Polychaete, Cirratuliformia, *Cossura*

## Abstract

Here, we report the complete mitochondrial genome of *Cossura pygodactylata* Jones 1956 (Annelida, Sedentaria, Cossuridae) (20,713 bp) comprised of two ribosomal RNAs, the ubiquitous set of 13 protein-coding genes (PCGs), 24 tRNAs, and a long AT-rich non-coding region. The order of PCGs and rRNAs is the same as in the previously reported genome of *Cossura aciculata* Wu & Chen 1977, but the order of tRNAs is different. Phylogenetic analysis of PCGs confirmed that Cossuridae is monophyletic and represents the sister group to Cirratulidae within Cirratuliformia.

## Introduction

**Figure 1.**
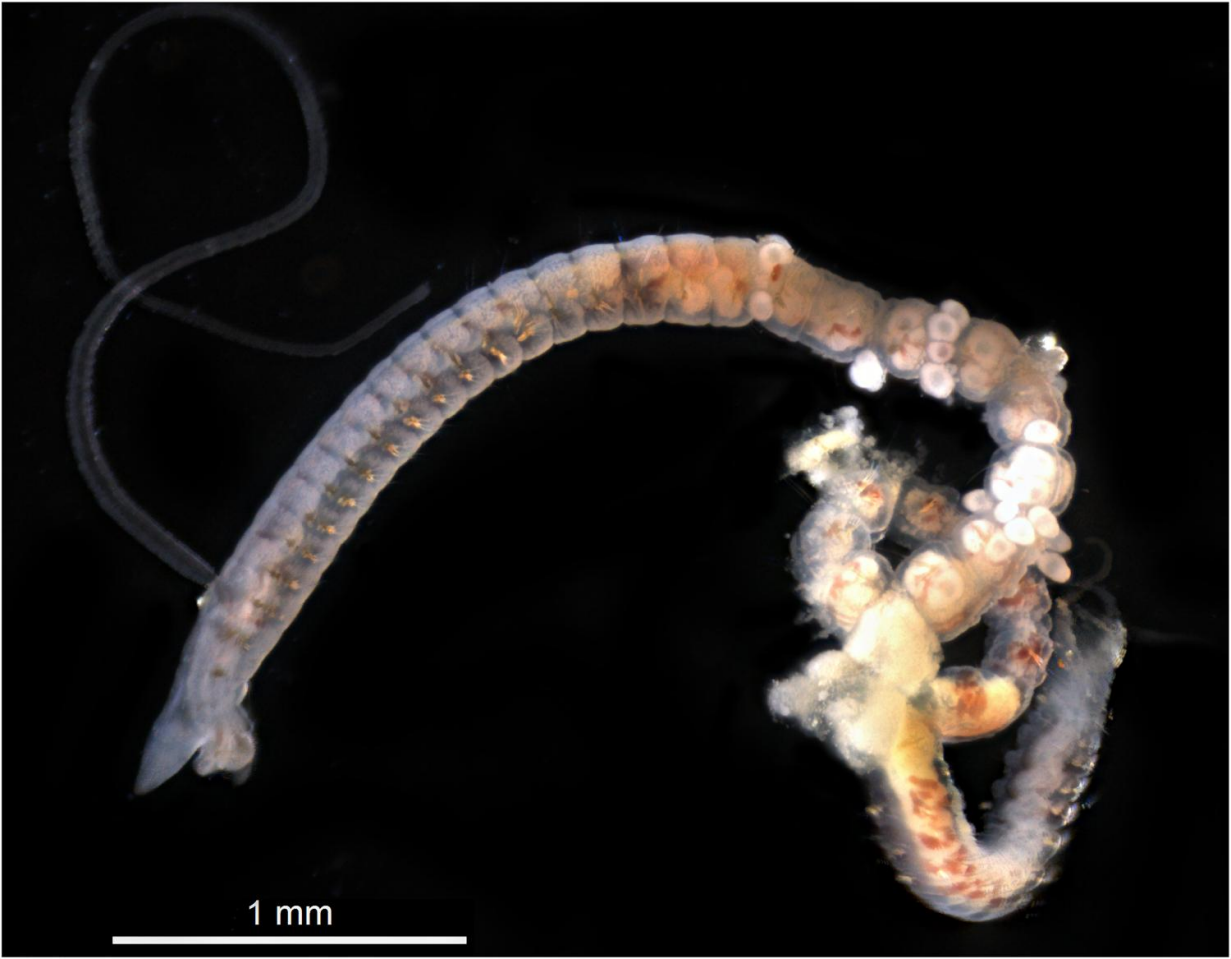
*Cossura pygodactylata*, specimen photographed by Anna Zhadan (August 2025), collected from Velikaya Salma Strait (Kandalaksha Bay, White Sea, Russia). Scale bar = 1 mm.

Cossuridae is a small, monotypic family of sedentary polychaetes that are often found in soft substrates and can dominate benthic communities (Grosse et al. 2021). They have a thin and elongated body, reduced parapodia, and a long branchial filament attached mid-dorsally. The phylogenetic affinity of Cossuridae remains uncertain. According to molecular data, Cossuridae (as well as Paraonidae) are grouped with the Cirratuliformia clade, but it is still unclear whether they truly belong to this clade (Zrzavý et al. 2009; Struck 2019). *Cossura pygodactylata* Jones 1956 (type locality San Francisco Bay, California) (Figure 1) is a widespread species inhabiting a broad range of depths and ecological niches, suggesting the possibility of cryptic speciation (Grosse et al. 2021). In this study, we present the mitochondrial genome of *Cossura pygodactylata* from the White Sea.

## Materials and methods

### Sample collection

Individuals were collected in 2016 and 2023 using a hyperbenthic sled and grab samplers from soft-sediment habitats at depths of 35–43 m in Velikaya Salma Strait (66.5°N, 33.2–33.3°E), Kandalaksha Bay, White Sea, Russia (see detailed information for each individual in Table S1). Samples were sieved through a 250-μm mesh, sorted under a stereomicroscope, and preserved in either 96% ethanol or RNAlater^®^. All specimens sequenced in this study were deposited at the collection of the White Sea branch of the Zoological Museum of Moscow State University (http://wsbs-msu.ru; Nikolai Neretin, nneretin@wsbs-msu.ru; vouchers ZMMU WS (Neretin et al. 2026)).

### DNA extraction, mitochondrial genome sequencing, assembly, and annotation

DNA was extracted from three samples (specimen vouchers ZMMU WS31492, ZMMU WS31493, and ZMMU WS31490) using a Diatom DNA kit (Isogen, Utrecht, The Netherlands). Here and further, all kits were used according to the manufacturer’s protocols and recommendations.

Whole genome DNA libraries for samples WS31492 and WS31493 were prepared separately using the NEBNext Ultra II DNA Library Prep Kit by New England Biolabs (NEB, Ipswich, MA) and sequenced on an Illumina HiSeq 4000 platform with a paired-end read length of 150. *De novo* contigs were assembled from trimmed reads (here and further trimming was performed using Trimmomatic (v.0.39) (Bolger et al. 2014)) with SPAdes (v.3.15.3) (Bankevich et al. 2012), and seven non-overlapping contigs were aligned to the *Orbinia latreillii* mitochondrial genome (GenBank ID: AY961084) using Mauve 20150226 (Darling et al. 2004) and MUMmer3.23 (Kurtz et al. 2004). However, these seven contigs had low coverage by the original reads and therefore cannot be considered reliable. Based on these contigs, we designed seven sets of primers for the long-range PCR, with amplicon length in 6–9 kb range (primers are listed in Table S2).

Using these long-range PCR primers, we amplified mitochondrial DNA from sample WS31490 with the Phanta Max Super-Fidelity DNA Polymerase (Vazyme, Nanjing, China) under the following conditions: 3 min at 95 °C; 35 cycles: 15 s at 95 °C, 15 s at 57 °C, 5 min at 72 °C; 5 min at 72 °C. Then, DNA library was prepared using the NEBNext Ultra II DNA Library Prep Kit by New England Biolabs (NEB, Ipswich, MA), and sequenced on an Illumina MiniSeq platform with a paired-end read length of 150.

The complete circular mitochondrial genome was *de novo* assembled from trimmed Illumina reads from sample WS31490 (1,167,217 reads in total) with the combination of SPAdes (v.3.15.3) (Bankevich et al. 2012) and NOVOPlasty (v.4.3.5) (Dierckxsens et al. 2017). Partial mitochondrial sequence from SPAdes assembly was used as a seed for NOVOPlasty. Mitochondrial genes were annotated using the MITOS2 software (Arab et al. 2017; Donath et al. 2019), and annotation was manually refined.

### RNA sequencing

RNA was extracted from one sample (specimen voucher ZMMU WS31506) using an ExtractRNA kit (Evrogen, Moscow, Russia). cDNA library was prepared using the NEBNext Ultra II RNA Library Prep Kit for Illumina (NEB, Ipswich, MA), and sequenced on Illumina NextSeq500 with a paired-end read length of 150. Trimmed reads were mapped to the reference mitochondrial genome with bowtie2 (v.2.5.4) (Langmead and Salzberg 2012), the maximum read depth equaled 8003 read bases per map position.

### Phylogenetic analysis

For phylogenetic analysis, protein sequences of 12 protein-coding genes (PCGs) (excluding ATP-synthase 8 subunit) of the assembled mitochondrial genome and 10 other species from Cirratuliformia suborder, downloaded from GenBank, were individually aligned using MAFFT v7.526 (Kuraku et al. 2013; Katoh et al. 2019). The following alignments were then concatenated into one alignment, and stable phylogenetic positions were chosen using Gblocks (v.0.91b) (Castresana 2000). The maximum-likelihood tree was reconstructed based on 3014 amino acid sites using the IQtree (v.2.4.0) (Kalyaanamoorthy et al. 2017; Hoang et al. 2018; Minh et al. 2020) with a model mtZOA + F + I + G4 and 1000 nonparametric bootstrap replicates. *Loimia arborea* and *Pista cristata* from the Terebellidae family were used as an outgroup.

## Results

**Figure 2.**
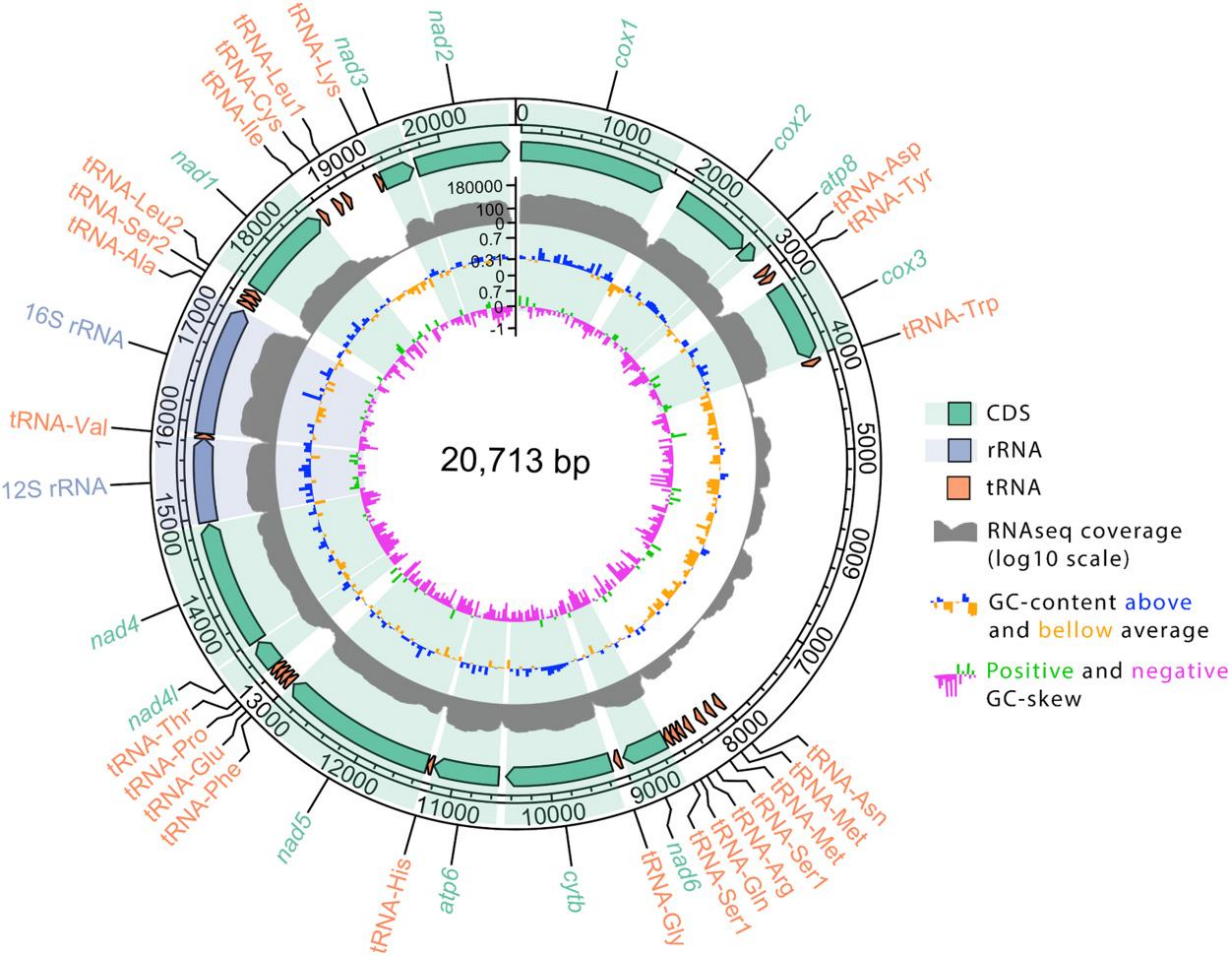
Mitochondrial genome of *Cossura pygodactylata*. Genome map, where the outer track represents the boundaries of standard mitochondrial PCGs, tRNAs, and rRNAs (green, orange, and blue arrows, respectively); sectors corresponding to PCGs and rRNAs are highlighted in green and blue. The arrow direction corresponds to the direction of transcription. Log10-scaled coverage of transcriptomic reads is shown in grey. GC content is shown in blue (above average) and yellow (below average); average GC content is 31%. GC skew index is shown in green (≥0) and pink (<0).

The complete mitochondrial genome of *Cossura pygodactylata* (ZMMU WS31490) is 20,713 bp long, with a GC content of 31%, and GC skew ((G − C)/(G + C)) of −0.24. The minimum and average coverage depths of the assembly were ×33 and ×4280 (Figure S1). The annotated genome contains all standard mitochondrial genes on the same strand: 13 PCGs, 24 tRNAs (tRNA-Met and tRNA-Ser1 genes are duplicated), and two rRNAs (Figure 2). To identify transcript boundaries and improve gene annotation, we also sequenced the transcriptome of *C. pygodactylata*. The RNAseq coverage for individual genes is shown as a separate track on Figure 2. There is a long (3899 bp) AT-rich non-coding region between tRNA-Trp and tRNA-Asn genes, with flanking PCGs being *cox3* and *nad6*; this region has lower coverage by the genomic (Figure S1) and transcriptomic (Figure 1) reads. The genome is ∼2000 bp longer than the published mitochondrial genome of *Cossura aciculata* (PV151471), which is 18,410 bp long. While the order of PCGs and rRNAs is the same, there are differences in placement and order of tRNAs (Figure S2).

**Figure 3.**
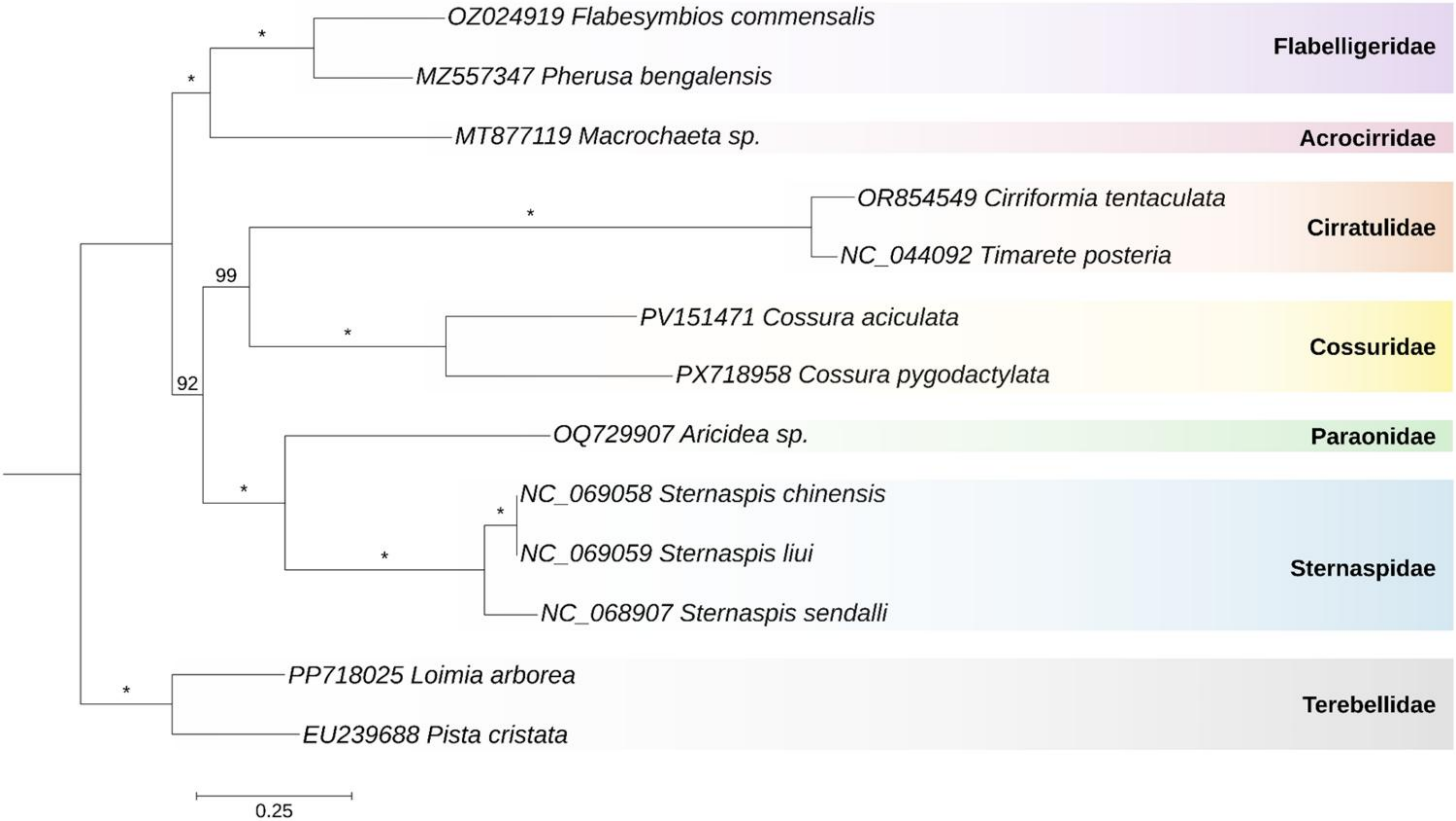
Phylogeny of the Cirratuliformia. Maximum-likelihood phylogenetic tree of the Cirratuliformia suborder and two species from the Terebellidae family as an outgroup. The tree is constructed based on concatenated alignments of amino acid sequences of 12 PCGs (all standard mitochondrial genes except for the ATP8). Bootstrap support values are shown at each node; asterisks indicate 100% support. Sequences from GenBank included in this tree are: *Flabesymbios commensalis*, OZ024919 (unpublished); *Pherusa bengalensis*, MZ557347 (unpublished); *Macrochaeta sp*., MT877119 (Sevigny et al. 2021); *Cirriformia tentaculata*, OR854549 (unpublished); *Timarete posteria*, NC_044092 (Kim et al. 2019); *Cossura aciculata*, PV151471 (unpublished); *Aricidea sp*., OQ729907 (Struck et al. 2023); *Sternaspis chinensis*, NC_069058 (unpublished); *Sternaspis liui*, NC_069059 (unpublished); *Sternaspis sendalli*, NC_068907 (unpublished); *Loimia arborea*, PP718025 (unpublished); *Pista cristata*, EU239688 (Zhong et al. 2008).

Maximum-likelihood phylogenetic tree of the Cirratuliformia suborder (Figure 3) resolves *C. pygodactylata* as sister to *C. aciculata* (with 100% bootstrap support). According to the tree, Cossuridae is sister to Cirratulidae (99% support), and the Cossuridae + Cirratulidae clade is sister to the Paraonidae + Sternaspidae clade (92% support). The broader (Cossuridae + Cirratulidae + Paraonidae + Sternaspidae) clade is sister to the Flabelligeridae + Acrocirridae clade (100% support).

## Discussion and conclusions

This study presents the first complete mitochondrial genome of *C. pygodactylata* (PX718958; 20,713 bp), and second mitogenome in the Cossuridae family, after *C. aciculata*.

Phylogenetic analysis confirms the monophyly of Cossuridae, with both *Cossura* species forming a distinct clade. Morphologically, these species differ significantly: *C. pygodactylata* possesses capillary chaetae along the entire body, whereas *C. aciculata* bears abdominal spines (a character previously used to place it in the separate genus *Cossurella* (Hartman 1976)).

The structure of the Cirratuliformia suborder differs from that reported in previous studies. Our analysis shows that Cossuridae is sister to Cirratulidae, while earlier works based on molecular and morphological data recovered *Cossura* as either sister to Paraonidae, or, alternatively, placed Cossuridae and Paraonidae as presumable sister clades to all other Cirratuliformia (Zrzavý et al. 2009; Struck 2019). These findings were based either on morphology and sequences of six genes (18S, 28S, and 16S rRNA, EF1α, H3, COI) (Zrzavý et al. 2009) or on transcriptomic analysis (Struck 2019). Interestingly, *Cossura* was historically classified within Cirratulidae prior to the establishment of the family Cossuridae (Day 1963). Cossuridae shares with Cirratulidae the following morphological characteristics: prostomium without appendages, parapodia lacking lobes, and capillary chaetae with flattened blades (Day 1963). These characters are most probably symplesiomorphies, as they are found in other families belonging to Cirratuliformia. A possible morphological synapomorphy of these two families could be the presence of long branchial appendages, segmental and paired in Cirratulidae, and a single, unpaired branchial filament in Cossuridae.

## Supplementary Material

TableS1.csv

Supplementary.docx

## Data Availability

The genome sequence data that support the findings of this study are openly available in GenBank of NCBI at https://www.ncbi.nlm.nih.gov/ under the reference number PX718958. The associated BioProject, SRA, and BioSample numbers are PRJNA1346847, SRR36928810, and SAMN54769951, respectively. RNA-Seq raw sequencing data available under BioProject, SRA, and BioSample numbers are PRJNA1346847, SRX30870365, and SAMN52842157, respectively.
