# Supplementary material for: Mitochondrial genome of Cossura pygodactylata Jones 1956 (Annelida: Sedentaria) from the White Sea
Source: Mitochondrial DNA B Resour. 2026 May 11;11(6):734–8. doi: 10.1080/23802359.2026.2670063 (PMC13162558; doi:10.1080/23802359.2026.2670063)
Supplement: Supplementary.docx [file TMDN_A_2670063_SM9677.docx]

### **Table S2.** List of primers used for the Long-Range PCR amplification of the mitochondrial genome of *Cossura pygodactylata*.

| **Primer_name** | **Primer_sequence** |
| --- | --- |
| lr3_forward | caacaggattccacggtctt |
| lr3_reverse | atccaagggcaagacttcct |
| lr4_forward | atggaccatcctccccttac |
| lr4_reverse | aatattgagcctgcaatggg |
| lr5_forward | ccctaataagcttgggggag |
| lr5_reverse | cctttttcaacgagagcgac |
| lr6_forward | accaatcgtattggagacgc |
| lr6_reverse | acgtcggtctgaactcagct |
| lr7_forward | cgcccctatgcagtattgat |
| lr7_reverse | tccagtaactcccccaactg |
| lr8_forward | gtcgctctcgttgaaaaagg |
| lr8_reverse | cctgggcaggtagtggttaa |
| lr9_forward | gctttacgcaagccctactg |
| lr9_reverse | aagaccgtggaatcctgttg |


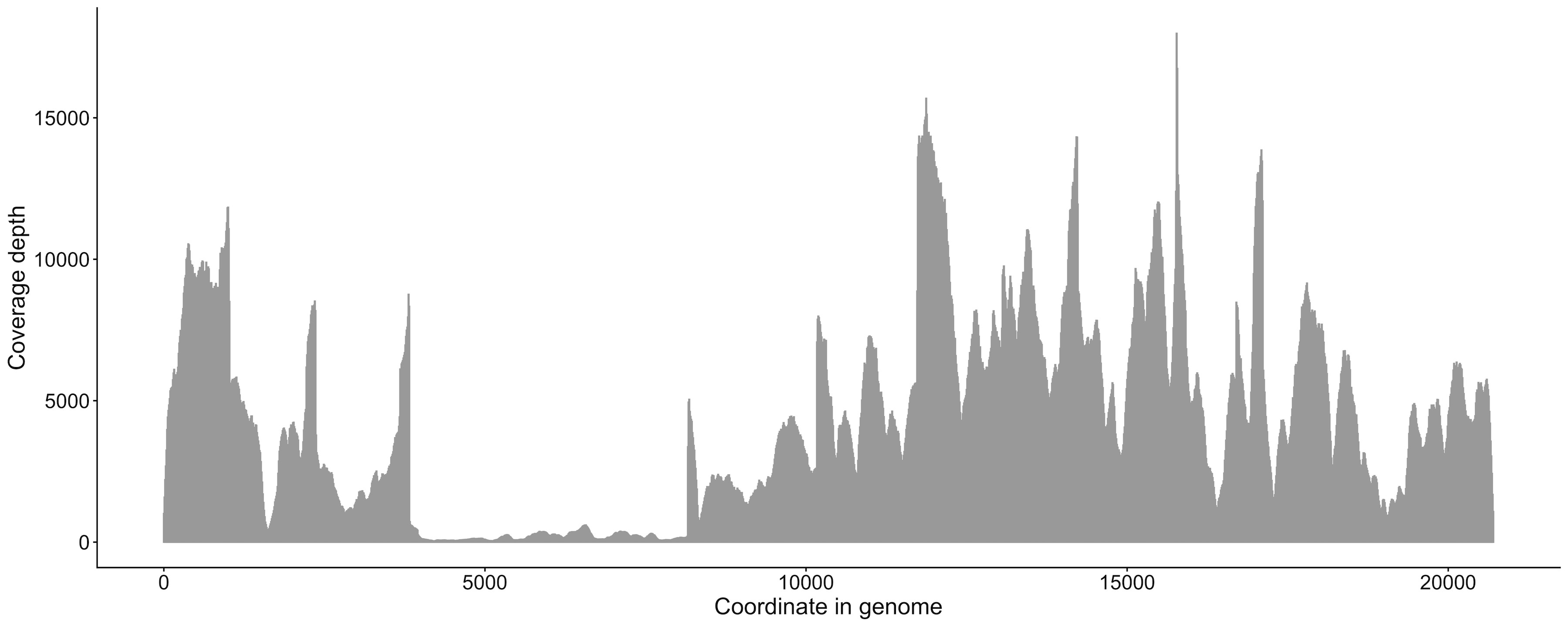


### **Figure S1.** Coverage depth of the assembled *Cossura pygodactylata* mitogenome.


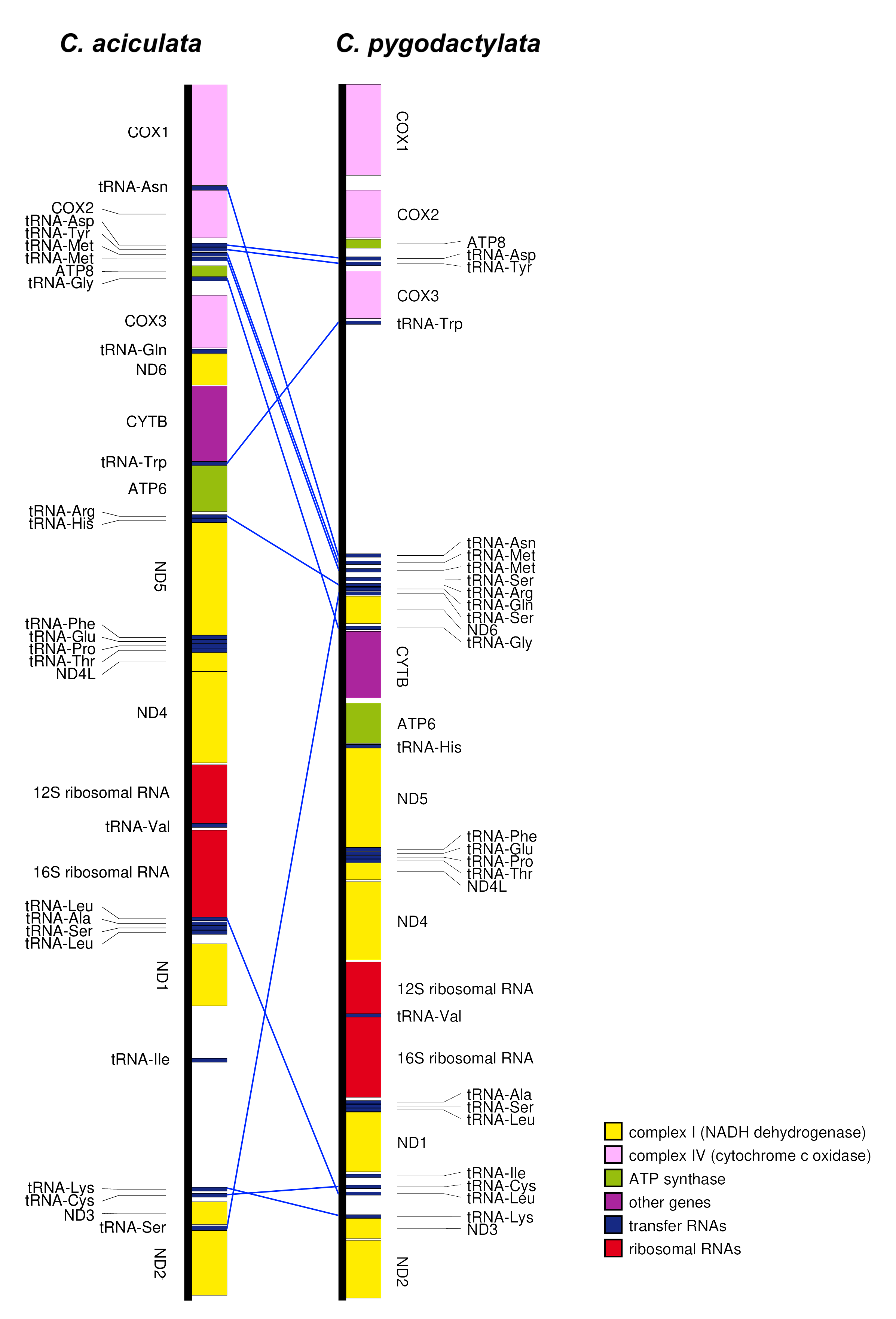


### **Figure S2.** Differences in tRNA order between *C. aciculate* (left) and *C. pygodactylata* (right) mitogenomes.
